# The ambiguous “internal carotid artery”–Ultrasound diagnosis of congenital absence of the internal carotid artery: A case report and review of the literature

**DOI:** 10.1097/MD.0000000000035016

**Published:** 2023-09-08

**Authors:** Jing Ning, Gang Zhong, Xiangdang Long, Juanjuan Xie, Kun Ao, Fang Liu, Mengyi Huang, Yu Zhuo, Qiaorong Li, Qiongli Wen, Qiuyi Di

**Affiliations:** a Department of Ultrasound, The First-Affiliated Hospital of Hunan Normal University (Hunan Provincial People’s Hospital), Changsha, China; b Department of Radiology, The First-Affiliated Hospital of Hunan Normal University (Hunan Provincial People’s Hospital), Changsha, China; c Department of Neurosurgery, The First-Affiliated Hospital of Hunan Normal University (Hunan Provincial People’s Hospital), Changsha, China.

**Keywords:** aneurysm, case report, congenital absence of the internal carotid artery, diagnosis, ultrasound

## Abstract

**Rationale:**

Congenital anatomical variation of internal carotid artery (ICA) rarely occurs, and congenital absence of the ICA is even rarer. Few reports are available on the diagnosis of congenital absence of the ICA by carotid doppler ultrasound (CDUS), and most cases have been identified by computed tomographic angiography (CTA) or digital subtraction angiography (DSA).

**Patient concerns:**

A 61-year-old male was admitted to our hospital due to dizziness for more than half a month. He was hypertensive and had been drinking and smoking for many years.

**Diagnoses:**

The patient was diagnosed by carotid doppler ultrasound with congenital absence of the right ICA, confirmed by CTA and DSA. A nodular aneurysm in the anterior communicating artery was observed by CTA and DSA.

**Interventions:**

After relevant preoperative examinations were performed, the patient underwent right craniotomy and clipping of the aneurysm under general anesthesia 8 days after admission.

**Outcomes:**

The patient recovered well after surgery and no relapses has been observed.

**Lessons:**

Congenital absence of the ICA is rare and usually diagnosed by CTA or DSA in clinical practice. If radiologists do not have adequate knowledge about the associated ultrasonic characteristics, a missed diagnosis may occur. As a noninvasive and rapid screening tool for cervical vascular diseases, carotid doppler ultrasound offers a new approach for the diagnosis of congenital absence of the ICA.

## 1. Introduction

The internal carotid artery (ICA) is one of the most important arteries of the brain and the most stable blood vessels in the human body. Congenital anatomical variation rarely occurs, and congenital absence of the ICA is even rarer, with an incidence of <0.01%.^[1–3]^ Patients with congenital absence of the ICA often have no obvious clinical symptoms; therefore, they are often discovered by chance during physical examination. However, this pathology is often accompanied by the formation of an intracranial aneurysm,^[1]^ which may lead to serious complications, such as aneurysm rupture and bleeding or even death. Therefore, early and accurate diagnosis of congenital absence of the ICA is an important measure to prevent stroke.

More than 300 cases have been found by radiology since the first case published in 1787 by Tode,^[1]^ while very few^[4,5]^ have been diagnosed by carotid doppler ultrasound (CDUS). CDUS, the most commonly used screening instrument for cervical vascular diseases, allows dynamic observation of the courses, lumen and wall structures of the carotid arteries; provides information about the blood flow velocity and Doppler spectrum; and facilitates the evaluation of hemodynamic changes and the diagnosis of congenital dysplasia. Congenital absence of the ICA is rare and usually diagnosed by computed tomographic angiography (CTA) or digital subtraction angiography (DSA). If radiologists do not have adequate knowledge about the associated ultrasonic characteristics, a missed diagnosis may occur.

## 2. Case report

A 61-year-old male presented to the internist’s office complaining of dizziness for more than half a month. He was hypertensive and had been drinking and smoking for many years. Neurological examination did not reveal significant sign. CDUS performed at an outside hospital showed no abnormality.

**Figure 1.**
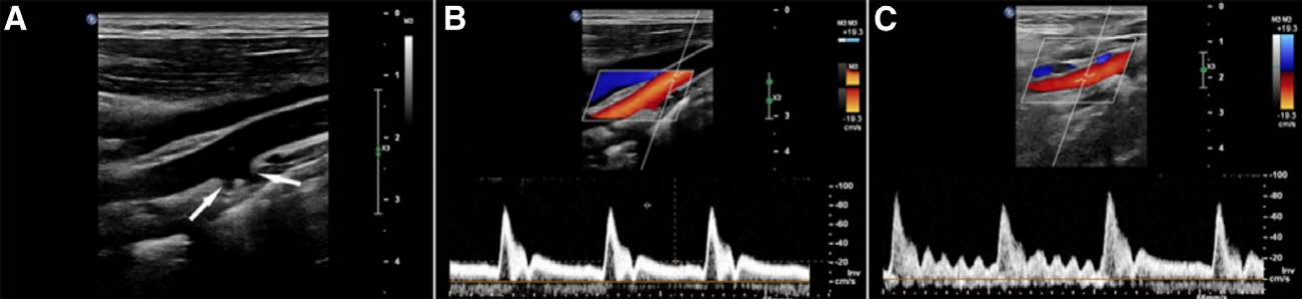
(A) “Bifurcation” of the RCCA - a directly continuing “ICA” and two adjacent small blood vessel branches (arrows). (B) Pulsed doppler shows that the blood flow velocity and Doppler spectrum of the RCCA are normal. (C) Pulsed doppler shows an obvious anterograde diastolic blood flow spectrum of the right “ICA” and a typical “saw-tooth” spectral waveform during diastole under the temporal tap maneuver. ICA = internal carotid artery, RCCA = right common carotid artery.

CDUS conducted at our hospital revealed a “bifurcation” containing a directly continuing “ICA” and 2 adjacent small blood vessel branches in the right common carotid artery (RCCA) (Fig. 1A). The RCCA was maldeveloped with normal blood flow velocity and Doppler spectrum (Fig. 1B). The right “ICA” showed a high-resistance Doppler spectrum and a “saw-tooth” appearance of the spectral waveform during diastole under the temporal tap maneuver (Fig. 1C). After tracing the courses of the 2 small blood vessels, we confirmed that the right “ICA” was actually the right external carotid artery. Therefore, congenital absence of the ICA was considered.

**Figure 2.**
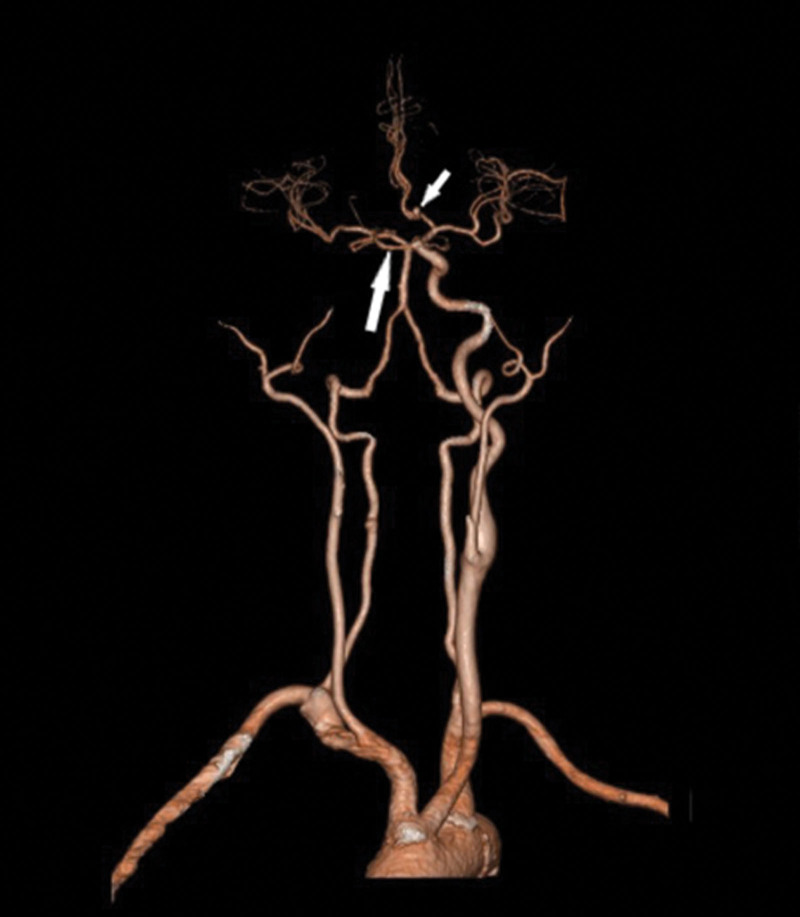
The RCCA is thinner than the LCCA and continues directly to the RECA. The LICA connects with the RMCA via an intercavernous branch between the cavernous sinuses (long arrow), and the LACA connects with the RACA via the ACoA, showing an ACoA aneurysm (short arrow). ACoA = anterior communicating artery, LACA = left anterior cerebral artery, LCCA = left common carotid artery, LICA = left internal carotid artery, RACA = right anterior cerebral artery, RCCA = right common carotid artery, RECA = right external carotid artery, RMCA = right middle cerebral artery.

**Figure 3.**
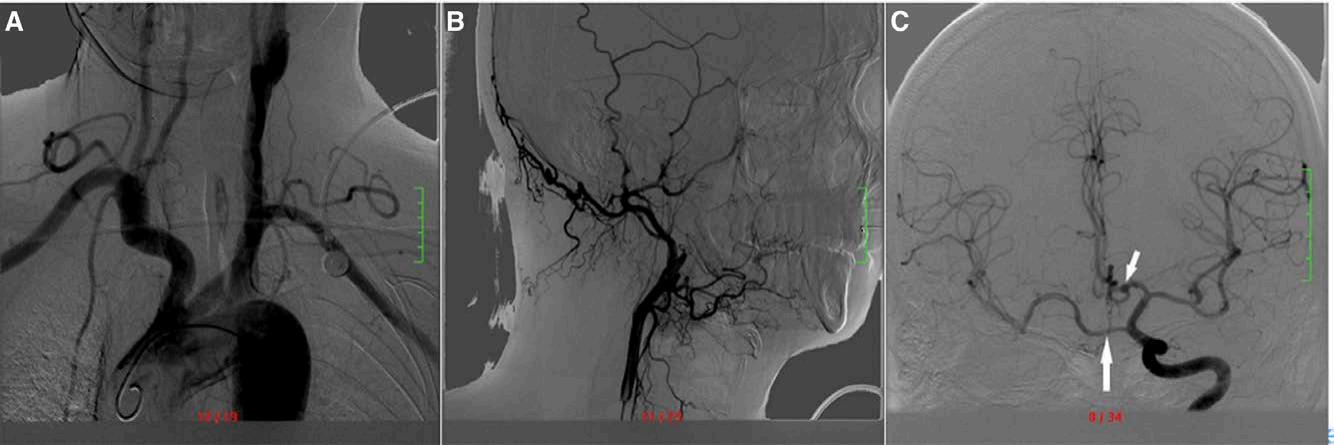
(A) The RCCA is obviously thinner than the LCCA, and no bifurcated structure is evident. (B) The RCCA continues directly to the RECA. (C) The RMCA is supplied by the LICA via the intercavernous branch between the cavernous sinuses (long arrow), and the RACA is supplied by the LACA via the ACoA, showing an ACoA aneurysm (short arrow). ACoA = anterior communicating artery, LACA = left anterior cerebral artery, LCCA = left common carotid artery, LICA = left internal carotid artery, RACA = right anterior cerebral artery, RCCA = right common carotid artery, RECA = right external carotid artery, RMCA = right middle cerebral artery.

CTA (Fig. 2) performed that the right ICA was absent, the RCCA had no obvious bifurcation, the left common carotid artery and the left ICA (LICA) were thick, the right anterior cerebral artery (RACA) A1 was absent, the RACA originated from the left ACA (LACA), and the right middle cerebral artery (RMCA) originated from a LICA branch. A nodular aneurysm with a diameter of approximately 3.7 mm could be observed in the anterior communicating artery (ACoA). DSA (Fig. 3) revealed an ACoA aneurysm. The right internal carotid artery (RICA) was not developed, the RACA was supplied by the LACA through the ACoA, and the RMCA was supplied by the LICA through an anastomotic branch between the cavernous sinuses.

**Figure 4.**
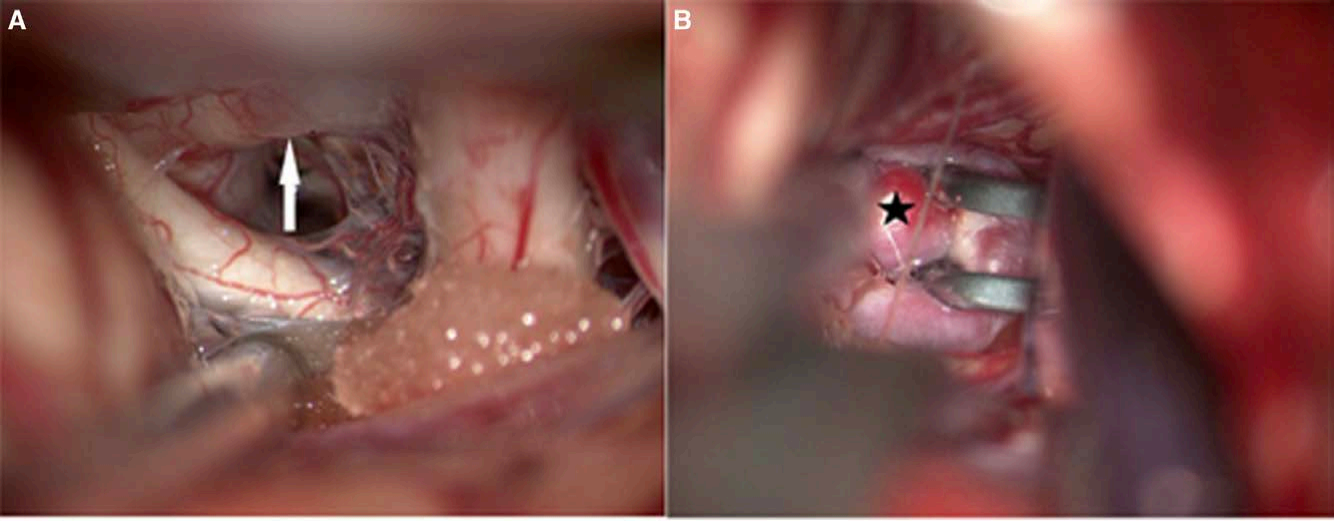
(A) The anastomotic branch between the cavernous sinuses (arrow) originates from the LICA and travels under the dura mater and above the tuberculum sellae. (B) The ACoA aneurysm (asterisk). ACoA = anterior communicating artery, LICA = left internal carotid artery.

After relevant preoperative examinations were performed, the patient underwent right craniotomy and clipping of the aneurysm under general anesthesia 8 days after admission. During the operation, an aneurysm measuring approximately 4 × 3 mm was observed in the ACoA complex of the brain, and the RICA was not explored. The LICA was observed to send out an intercavernous branch that connected with the RMCA above the tuberculum sellae and below the dura mater (Fig. 4). So far, the patient recovered well after surgery and no relapses has been observed.

## 3. Discussion

At present, the pathogenesis of congenital absence of the ICA is unclear. The disease is possibly related to arrested development or secondary degeneration of the ICA during embryonic development.^[1]^ Some studies^[1–3]^ have reported that the incidence of congenital absence of the ICA is <0.01%, with unilateral absence being more common, and the ratio of left to right absence is approximately 3:1. Bilateral absence accounts for <10% of all cases of congenital absence of the ICA. Patients with congenital absence of the ICA often have no clinical symptoms due to compensation by the collateral circulation of the circle of Willis. Some patients may experience non characteristic symptoms such as tinnitus, dizziness and headache, and a few patients may be admitted to the hospital due to aneurysm rupture or epilepsy.^[6]^ Lie^[7]^ divided congenital absence of the ICA into 6 types according to the compensatory pathways of collateral circulation. In Type A (unilateral absence of the ICA), the most common type encountered in clinical practice, the affected anterior cerebral artery (ACA) is supplied by the contralateral ACA via the ACoA, and the affected middle cerebral artery (MCA) is supplied by the ipsilateral posterior cerebral artery. In Type B (unilateral absence of the ICA), the affected ACA and MCA are supplied by the contralateral ACA via the ACoA. In Type C (bilateral absence of the ICA), the bilateral ACAs and MCAs pass through the posterior communicating artery to supply blood from the posterior cerebral circulation. In Type D (unilateral absence of the ICA), the siphon section of the affected ICA is supplied by the contralateral ICA via the formation of an intercavernous branch between the cavernous sinuses. In Type E, the bilateral small ACAs are supplied by the bilateral maldeveloped ICAs, and the bilateral MCAs are supplied by the posterior cerebral artery via the posterior communicating artery. In Type F, the ACA and MCA on the affected side are supplied by anastomosis of the external carotid artery (ECA)-internal maxillary artery to the skull base, that is, the skull base microvascular network. This case corresponds to Type D in the Lie classification which is very rare. The RACA A1 was absent, the RACA was supplied by the LACA via the ACoA, and the RMCA was supplied by the LICA through the intercavernous branch of the cavernous sinuses. According to the study results of Jesse et al,^[8]^ the incidence of intracranial aneurysms was approximately 2% to 4% in the general population but as high as 25% to 43% in patients with congenital absence of the ICA, which might be caused by congenital dysplasia of the vascular wall or abnormal hemodynamics.^[1,9]^ Therefore, early and accurate diagnosis of congenital absence of the ICA and regular monitoring of the formation and development of intracranial aneurysms are important for preventing cerebral hemorrhage and subarachnoid hemorrhage.^[10]^

CDUS is the most commonly used screening tool for cervical vascular diseases.^[11]^ It offers the advantages of high resolution, high safety, convenience and low cost and plays a significant role in the diagnosis of congenital dysplasia of the carotid artery. Congenital absence of the ICA is so rare that most publications in the literature are case reports, and most cases were diagnosed by CTA or DSA, while few were diagnosed by CDUS.^[1]^ Therefore, this pathology may be missed during ultrasonic examination. In the present case, CDUS conducted at an outside hospital indicated no abnormalities. During examination, we found that the patient’s RCCA was maldeveloped, but its blood flow velocity and Doppler spectrum were normal. When we observed a “bifurcation” of the RCCA, we mistakenly thought that the thicker blood vessel directly extending from the RCCA was the ICA. Pulsed Doppler showed that its resistance index was significantly higher than that of the LICA and slightly higher than that of the RCCA; therefore, it was suspected to be the ECA. The temporal tap maneuver elicited a “saw-tooth” appearance of the spectral waveform. Careful scanning revealed 2 adjacent small blood vessel branches at the “bifurcation.” By tracing their courses, we observed that the first branch was the superior thyroid artery, and the other branch extended toward the side of the head. Therefore, we thought that no right ICA was present and that the RCCA continued directly to the ECA.

When absence of the ICA is suspected on ultrasound, it should be differentiated from ICA occlusion. Occlusion of the ICA is most often caused by arteriosclerosis, but it can also be observed in cases of arteritis, cardiogenic embolism and so on. Ultrasound can not only show the wall structure of the occluded ICA but also be used to explore the stump of the ICA.^[12]^ Transthoracic echocardiogram can be performed when cardiogenic embolism is considered. Kaya et al^[4]^ suggested that maldeveloped common carotid arterie (CCA) on the affected side was an important clue in the ultrasonic diagnosis of congenital absence of the ICA. Yilmaz et al^[5]^ found that most patients with ICA occlusion showed a reduced systolic peak velocity of the CCA on the affected side, and the diastolic velocity was significantly reduced, even the Doppler spectrum was reversed. However, the systolic peak velocity of the CCA in cases of congenital absence of the ICA was normal. Therefore, we would not consider ICA occlusion when the ICA is not present and the CCA on the affected side shows a low-resistance Doppler spectrum. In this case, the RCCA was maldeveloped, and the Doppler spectrum, blood flow velocity and resistance index of the bilateral CCAs were very similar, without the obvious wall structure of the RICA. Therefore, the RICA was considered to be absent rather than occluded. In addition, because the ICA forms earlier than the skull base, CT of the skull base can also assist in the diagnosis of congenital absence of the ICA if the bony carotid canal is maldeveloped or not developed.^[9]^

As a noninvasive and rapid screening tool for cervical vascular diseases, CDUS offers a new approach for the diagnosis of congenital absence of the ICA. When maldevelopment of the CCA is found on 1 side and pulsed Doppler shows that the blood flow velocity and Doppler spectrum are normal and the ipsilateral ICA is not present, congenital absence of the ICA should be considered.

## Author contributions

**Resources:** Juanjuan Xie, Kun Ao, Fang Liu, Mengyi Huang, Yu Zhuo, Qiaorong Li, Qiongli Wen, Qiuyi Di.

**Writing – original draft:** Jing Ning, Gang Zhong.

**Writing – review & editing:** Xiang Dang Long.
